# Risk of Sperm Disorders and Impaired Fertility in Frozen–Thawed Bull Semen: A Genome-Wide Association Study

**DOI:** 10.3390/ani14020251

**Published:** 2024-01-13

**Authors:** Natalia V. Dementieva, Artem P. Dysin, Yuri S. Shcherbakov, Elena V. Nikitkina, Artem A. Musidray, Anna V. Petrova, Olga V. Mitrofanova, Kirill V. Plemyashov, Anastasiia I. Azovtseva, Darren K. Griffin, Michael N. Romanov

**Affiliations:** 1 Russian Research Institute of Farm Animal Genetics and Breeding—Branch of the L. K. Ernst Federal Research Centre for Animal Husbandry, Pushkin, 196601 St. Petersburg, Russia; artemdysin@mail.ru (A.P.D.); yura.10.08.94.94@mail.ru (Y.S.S.); nikitkinae@yandex.ru (E.V.N.); 13linereg@mail.ru (A.A.M.); anusha.82@mail.ru (A.V.P.); mo1969@mail.ru (O.V.M.); ase4ica15@mail.ru (A.I.A.); 2 Federal State Budgetary Educational Institution of Higher Education “St. Petersburg State University of Veterinary Medicine”, 196084 St. Petersburg, Russia; kirill060674@mail.ru; 3 School of Biosciences, University of Kent, Canterbury CT2 7NJ, UK; d.k.griffin@kent.ac.uk; 4 L. K. Ernst Federal Research Centre for Animal Husbandry, Dubrovitsy, 142132 Podolsk, Moscow Oblast, Russia

**Keywords:** frozen-thawed bull semen, genome-wide association study, single nucleotide polymorphism, sperm abnormalities, Holstein cattle

## Abstract

**Simple Summary:**

This study tackles the genetic aspects of the risk of sperm damage and related impaired fertility when handling frozen–thawed bull semen for artificial insemination. To this end, we performed genomic association analysis to identify relevant genetic markers and candidate genes associated with various abnormalities in frozen–thawed Holstein cattle sperm. The results provide important insights into the molecular mechanisms underlying sperm morphology and abnormalities after cryopreservation. Further research is needed to explore causative genetic variants and implement these findings to improve animal reproduction and breeding.

**Abstract:**

Cryopreservation is a widely used method of semen conservation in animal breeding programs. This process, however, can have a detrimental effect on sperm quality, especially in terms of its morphology. The resultant sperm disorders raise the risk of reduced sperm fertilizing ability, which poses a serious threat to the long-term efficacy of livestock reproduction and breeding. Understanding the genetic factors underlying these effects is critical for maintaining sperm quality during cryopreservation, and for animal fertility in general. In this regard, we performed a genome-wide association study to identify genomic regions associated with various cryopreservation sperm abnormalities in Holstein cattle, using single nucleotide polymorphism (SNP) markers via a high-density genotyping assay. Our analysis revealed a significant association of specific SNPs and candidate genes with absence of acrosomes, damaged cell necks and tails, as well as wrinkled acrosomes and decreased motility of cryopreserved sperm. As a result, we identified candidate genes such as *POU6F2*, *LPCAT4*, *DPYD*, *SLC39A12* and *CACNB2*, as well as microRNAs (*bta-mir-137* and *bta-mir-2420*) that may play a critical role in sperm morphology and disorders. These findings provide crucial information on the molecular mechanisms underlying acrosome integrity, motility, head abnormalities and damaged cell necks and tails of sperm after cryopreservation. Further studies with larger sample sizes, genome-wide coverage and functional validation are needed to explore causal variants in more detail, thereby elucidating the mechanisms mediating these effects. Overall, our results contribute to the understanding of genetic architecture in cryopreserved semen quality and disorders in bulls, laying the foundation for improved animal reproduction and breeding.

## 1. Introduction

Sperm disorders in frozen–thawed bull semen, widely used in artificial insemination (AI) technology, confer a risk of impaired fertility in cattle (*Bos taurus*; BTA). Semen cryopreservation is nonetheless a valuable and effective method for disseminating and preserving superior genetics in livestock production (e.g., [1,2,3,4,5]). It allows the use of AI to increase reproductive efficiency and genetic gain in livestock [6]. Sperm cryopreservation, however, also involves various physical and chemical stresses on sperm, such as osmotic shock, cold shock, oxidative stress and ice crystal formation, which can adversely affect morphology, motility and viability [7,8]. In turn, sperm disorders increase the risk of impaired sperm fertilizing ability, a major factor jeopardizing the sustainability of animal breeding [9]. Therefore, it is important to evaluate the quality and likely disorders of cryopreserved sperm before using it for AI or other assisted reproductive technologies.

One of the most important parameters of semen quality and its associated disorders is sperm morphology; this reflects its structural and functional integrity [10]. Sperm morphology can be influenced by various factors such as breed, age, nutrition, season, collection method and sperm processing [11]. Herewith, breed may have very little effect on sperm morphology, possibly due to high inbreeding in small breeds. For example, our previous study [3] also showed little influence of breed on sperm morphology. Abnormal sperm morphology can reduce the chances of fertilization [12] and embryo development; it can also increase the risk of transmitting genetic defects [13]. Morphology is therefore usually assessed during reproductive fitness examinations and control tests for disorders in semen samples. As a rule, various types of morphological defects are observed in the head, midpiece, tail and acrosome of spermatozoa [14].

The acrosome is a specialized organelle that covers the anterior part of the sperm head and contains enzymes (e.g., [15]) necessary for sperm to penetrate the *zona pellucida* of the egg. The acrosome undergoes a number of changes during the condensation and acrosome reaction that are necessary for successful fertilization [16]. Cryopreservation, however, may cause irreversible changes in acrosome structure and function, such as swelling, rupture, prolapse or premature reaction. These changes can impair the ability of sperm to bind and fuse with the egg membrane [17].

In addition to acrosome defects, cryopreservation can also cause other types of morphological abnormalities in the head, midpiece and tail of the sperm. For example, cryopreservation can lead to head anomalies such as microcephaly, roundness, asymmetry, shortening, pointedness or duplication [18]. Cryopreservation may also affect the integrity of the midpiece and its fit to the head [19]. In addition, it can damage the structure and function of the tail, causing curling, bending, tearing or detachment (e.g., [20,21,22]).

Sperm motility is another key parameter of sperm quality and disorders that is affected by cryopreservation. This parameter reflects the energy metabolism and fluidity of the sperm membrane. It is important for the movement of sperm through the female reproductive tract and for penetration into the egg. Sperm motility can be divided into the following different categories, based on the speed and direction of movement: (1) progressive motility, characterized by the percentage of sperm that move forward in a straight or slightly curved line; (2) non-progressive motility, referring to the percentage of spermatozoa that move in a circle or randomly; and (3) spermatozoa that do not move at all and are considered immotile [23]. Cryopreservation can impair sperm motility by affecting various aspects of sperm physiology. For example, it can change the structure of the lipid membrane and its fluidity and disrupt energy metabolism and ATP production [24]. In addition, cryopreservation can also induce oxidative stress and generate reactive oxygen species that can damage sperm membrane and DNA, as well as activate apoptosis and caspase pathways that can lead to gamete death or dysfunction [25].

Despite the availability of various methods for assessing sperm morphology and motility after cryopreservation, there is still a lack of consensus regarding the optimal criteria and threshold values for defining normal and abnormal sperm parameters [25]. In addition, information on the genetic factors influencing sperm morphology and motility in bulls after cryopreservation is quite limited. Previous studies have shown that sperm morphology and motility are heritable traits that can be influenced by genomic variations among certain species, breeds and even individuals [26,27,28]. Using genome-wide association studies (GWAS) based on single nucleotide polymorphism (SNP) markers [2,3,29,30,31], the identification of genomic regions and genes associated with these traits can provide valuable information about the molecular mechanisms underlying sperm quality and fertility. Several studies have examined genetic markers associated with semen quality and fertility in cattle using GWAS. For example, Abril-Parreño et al. [32] identified SNPs and candidate genes associated with field fertility and sperm quality in Holstein Friesian bulls before freezing. Kamiński et al. [33] found five significant markers on chromosome BTA6 associated with the integrity of sperm membranes in frozen–thawed semen of Holstein Friesian bulls. Also, Ramirez-Diaz et al. [34] found 10 genomic regions on eight chromosomes associated with total and progressive motility in Italian Holstein bulls. However, the genetic architecture of cryopreserved sperm with regard to quality and abnormalities is still largely unknown.

The purpose of this study was to conduct a high-density SNP genotyping assay and a GWAS methodology of sperm disorders in Holstein bulls after cryopreservation. The hypothesis is that there are genomic loci and candidate genes associated with sperm morphology and motility disorders in cryopreserved bovine semen. Specifically, the following sperm characteristics and defects were examined: absence of acrosomes, head abnormalities, damaged tails, midpiece abnormalities, swollen acrosomes, wrinkled acrosomes and progressive and total sperm motility. The purpose was to facilitate the development of genomic tools to improve male fertility in cattle and ultimately expedite breeding progress.

## 2. Materials and Methods

### 2.1. Animals and Sample Collection

The phenotypic data of 96 bulls were collected at Nevskoye OJSC (St. Petersburg, Russia). Their semen quality scores were assessed over the period of 2007 to 2018. The bulls were kept on a stock farm from the age of six months. Semen samples were collected from each bull using an artificial vagina. Sperm was collected at intervals of three to four days. Data for most bulls during the first three years of semen collection were analyzed. For bulls with poor semen quality, data were assessed prior to culling. An average of 151 (with the range of 49 to 215) ejaculates per bull were analyzed; the best sperm quality performance from each bull was taken for analysis.

### 2.2. Sperm Evaluation

Ejaculates were assessed for volume, concentration and percentage of live sperm. Frozen semen samples were thawed at 37 °C for 30 s, and sperm motility and morphology after thawing were assessed using computer-assisted sperm analysis (CASA, ArgusSoft Ltd., St. Petersburg, Russia; [35]) and an Axio Imager microscope (Carl Zeiss Microscopy GmbH, Jena, Germany). Motility parameters involved total and progressive motility. Morphological parameters included absence of acrosomes, swollen acrosomes, wrinkled acrosomes, head defects, cell neck defects and damaged tail defects. The analysis of morphological indicators of sperm quality was carried out according to the procedure for assessing animal sperm established for the Network Bioresource Collection of Farm Animals, Birds, Fish and Insects, Russian Research Institute of Farm Animal Genetics and Breeding (Pushkin, Russia; e.g., [3,36]). Cryopreserved and native semen samples were examined, although only data for cryopreserved sperm were used for the subsequent GWAS.

### 2.3. Genotyping Quality Control

For DNA extraction, 50 bull semen samples were taken. Genotypes obtained from the high-density BovineSNP50 v3 BeadChip (Illumina Inc., San Diego, CA, USA; [37,38]) were subjected to quality control using PLINK software [29]. SNPs with call rates below 95%, minor allele frequencies below 5%, or deviations from the Hardy–Weinberg equilibrium (*p* < 0.0001) were excluded from the analysis. In addition, animals with genotype detection rates below 95% were also removed. As a result, 45 samples with a complete assessment of sperm morphology were included in the analysis.

### 2.4. Genome-Wide Association Analysis

Genome-wide association analyses were performed using EMMAX software [30], which implements a mixed linear model that takes into account population structure and relatedness between individuals. The genomic linkage matrix was calculated using the VanRaden [39] method, based on the IBS (identity by state) matrix. The following model was used to estimate the effect of SNPs on each trait of sperm quality and disorder:

*Y* = *Xb* + *u* + *Br* + *e*,

where *Y* is a vector of phenotypes; *b* is a SNP effect; *X* is a design matrix of SNP genotypes; *u* is a vector of additive genetic effects that is supposed to have a normal distribution with a mean equal to zero and (co)variance *σ*^2^*aG*, where *σ*^2^*a* represents additive genetic variance and *G* is the genomic relationship matrix; *Br* is a breed effect; and *e* is a vector of random residual effects.

The significance threshold for SNP associations was determined by applying a Bonferroni correction, based on the number of effective independent tests estimated by the simpleM method in R software [40]. The Manhattan plots were generated using the qqman package in R to visualize the genome-wide association results [41]. SNPs that exceeded the significance threshold were annotated using the NCBI and Ensembl genomic browsers to identify nearby genes and their functions.

## 3. Results

Our GWAS results provide new insight into the genetic basis of cryopreserved bull sperm quality and its associated disorders. That is, we revealed significant associations between certain genetic variants and sperm quality traits related to acrosome integrity, sperm motility, head abnormalities and damaged tails and cell necks (midpieces). These data provide valuable information about the genetic architecture of cryopreserved sperm quality in bulls and examples of genomic locations of the suggested candidate genes associated with bull sperm disorders are shown in Table 1.

### 3.1. Sperm Head Abnormalities

#### 3.1.1. Absence of Acrosomes

**Figure 1:**
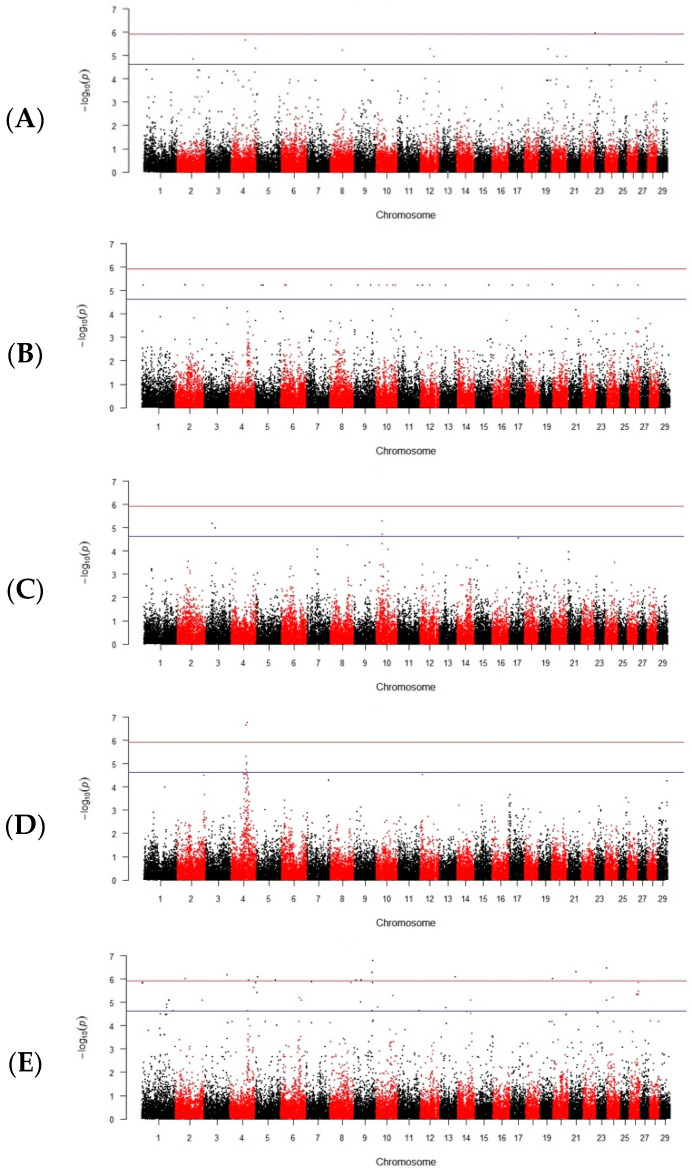

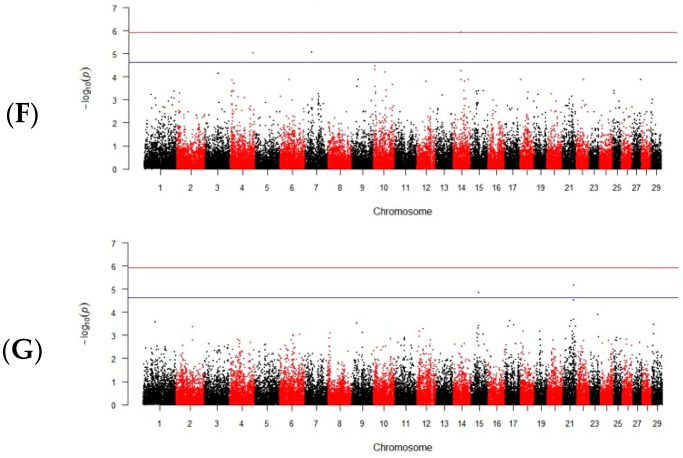
Manhattan plots for the sperm disorder indicators after cryopreservation: (**A**) absence of acrosomes, (**B**) abnormal head, (**C**) swollen acrosomes after cryopreservation, (**D**) wrinkled acrosomes, (**E**) damaged tails and cell necks, (**F**) progressive motility and (**G**) total motility.

The percentage of this sperm disorder observed in the present study was 2.4%. The subsequent GWAS investigation resulted in the identification of various significant SNPs associated with the absence of acrosomes after cryopreservation (Table 1, Supplementary Tables S1 and S2, Figure 1A). In all, 28 significant SNPs were located on BTA 2, 4, 8, 19, 20, 23, 24 and 29 and assigned to the corresponding candidate genes. The most significant candidate genes were *POU6F2* (POU class 6 homeobox 2), *MAP3K7* (mitogen-activated protein kinase kinase kinase 7), *TCF23* (transcription factor 23), *ABHD1* (abhydrolase domain containing 1), *SPDYA* (speedy/RINGO cell cycle regulator family member A) and *PPP1CB* (protein phosphatase 1 catalytic subunit beta).

#### 3.1.2. Sperm Head Abnormalities

The observed level of these head disorders was 2.4%. The appropriate genomic data obtained are summarized in Table 1, Supplementary Tables S1 and S2, and Figure 1B. Overall, there were 26 significant SNPs associated with the respective candidate genes, and these were localized on BTA 1, 2, 5, 6, 8–10, 12, 13, 15, 17–19, 22–24, and 26. *ORC4* (origin recognition complex subunit 4) and *GLRA3* (glycine receptor alpha 3) were the most significant genes of those associated with these disorder traits.

#### 3.1.3. Swollen Acrosomes

The level of this disorder was 2.9%. We found five significant SNPs on BTA 3, 4, and 10 that were significantly associated with the swollen acrosomes in the Holstein cattle sperm after cryopreservation (Table 1, Supplementary Tables S1 and S2, Figure 1C). Hereby, we identified two candidate genes, *LPCAT4* (lysophosphatidylcholine acyltransferase 4) and *DPYD* (dihydropyrimidine dehydrogenase), as well as two microRNAs, *bta-mir-137* and *bta-mir-2420*. These candidate genes and miRNAs may play a potential role in affecting cryopreserved sperm morphology and quality and, therefore, in causing the impaired male fertility in Holstein cattle.

#### 3.1.4. Wrinkled Acrosomes

The percentage of the wrinkled acrosome disorder in Holstein cattle sperm following its cryopreservation was 5%. We found four significant SNPs on BTA4 (Table 1, Supplementary Tables S1 and S2, Figure 1D). Accordingly, four genes were associated with this trait, including *IGFBP3* (insulin-like growth factor binding protein 3), *NPY* (neuropeptide Y), *MON2* (MON2 homolog, regulator of endosome-to-Golgi trafficking) and *STEAP1* (STEAP family member 1). Thus, the listed candidate genes may have a potential impact on sperm morphology and quality and, correspondingly, induce a lower bull fertility in Holstein cattle after cryopreservation.

### 3.2. Damaged Tails and Cell Necks

The level of this disorder accounted for 2.4%. We found 62 significant SNPs on BTA 1–15, 17–19, 21–24, and 26 (Table 1, Supplementary Tables S3 and S4, Figure 1E). They were associated with 24 genes that were uniquely associated with this trait and, to the best of our knowledge, have not been previously related to spermatogenesis abnormalities. Notably, most of the genes identified as being associated with damaged cell necks and tails were also associated with head abnormalities in the frozen–thawed bull spermatozoa established in this investigation.

### 3.3. Total and Progressive Motility

The percentage of this disorder was 86.7%. We detected a total of five SNPs that were found on BTA 4, 7, 14, 15, and 21 (Table 2, Figure 1F,G). There were seven candidate genes that may potentially affect the maintenance of sperm motility after the freeze–thaw process. Among them, the *FSCB* (fibrous sheath CABYR binding protein) gene was previously shown to have the highest expression in the testes [42].

## 4. Discussion

### 4.1. Genomic Associations with Sperm Head Disorders after Freezing

#### 4.1.1. Absence of Acrosomes

A significant SNP, BTB-00566744, was identified that was located on BTA4, being an intronic variant of the *POU6F2* gene. *POU6F2* belongs to the POU domain family of transcription factors and is known to regulate gene expression in various tissues and at various developmental stages [43]. Previous studies demonstrated that *POU6F2* is expressed in the testes and epididymis of mice and humans [44,45], assuming its involvement in spermatogenesis and sperm maturation. Moreover, knockout of this gene led to increased expression of prolactin [46]. Prolactin, in turn, significantly reduces the spontaneous acrosome reaction [47]. Our finding suggested that the determined SNP in *POU6F2* may alter its function, which is important for bovine spermatogenesis/sperm maturation, thereby affecting acrosome integrity.

Other candidate genes were also identified that may influence the absence of acrosomes after cryopreservation. Notably, a block of significant SNPs was found on BTA9 near the *MAP3K7* gene. MAP3K7 is a kinase that can activate MAPK8/JNK and MAP2K4/MKK4 pathways and, thus, plays a role in the response of cells to environmental stress. It was previously shown to be relevant to sperm maturation [48]. Similarly, three significant SNPs were found in a gene on BTA11, with *TCF23*, *ABHD1*, *SPDYA* and *PPP1CB* being identified as candidate genes potentially affecting acrosome formation. It is worth noting that a significant SNP, ARS-BFGL-NGS-37893, was associated with both *TCF23* and *ABHD1* (Supplementary Table S1). It has been found that these genes are expressed in the testes [49,50,51,52] and may be pivotal in maintaining acrosome integrity after freezing.

Our analysis also revealed a number of significant SNPs on chromosomes 15, 17, 23, 24 and 27, associated with genes involved in the activity of mineralocorticoid receptors (*NR3C2*, or nuclear receptor subfamily 3 group C member 2; [31,53]), transcription regulation (*ZNF26* and *ZNF84*, or zinc finger proteins 26 [54] and 84 [55]), phospholipid transport ATPase (*ATP8B1*, or ATPase phospholipid transporting 8B1; [56]) and in the self-renewal and meiosis of spermatogonial stem cells (*NRG1*, or neuregulin 1; [57]). Our findings suggest a potential role for these genes in controlling sperm quality and acrosome maintenance after cryopreservation. In addition, the candidate gene for CDC42EP2 (CDC42 effector protein 2) on BTA29 was identified as potentially involved in the organization of the actin cytoskeleton [58], which, in turn, is critical for maintaining the shape and integrity of germ cells as well.

The above candidate genes provide insight into the molecular mechanisms underlying acrosome integrity and sperm quality after freezing. It is important to note that, although our study identified genomic associations between specific genes and the absence of acrosomes after cryopreservation, further research is required to fully understand the functional implications of these associations. Also, the precise mechanisms by which these genes influence acrosome integrity and sperm quality require further study. In addition, potential interactions between these genes and other factors involved in spermatogenesis and sperm maturation need to be explored. To the best of our knowledge, our study is the first to conduct a comprehensive genomic survey of dairy bull semen properties associated with acrosome integrity after cryopreservation.

#### 4.1.2. Sperm Head Abnormalities Following Cryopreservation

We identified several candidate genes involved in various biological processes related to spermatogenesis, meiosis, cell polarity, signaling, gene expression and fertilization. These genes may underlie cryopreserved sperm head abnormalities that lead to impaired fertility in bulls.

One of the candidate genes we identified was *ORC4*, which encodes a subunit of the origin of replication recognition complex (ORC) involved in the initiation of DNA replication [59]. ORC4 plays a role in asymmetric meiotic division [60], and its oligomerization is required for polar body extrusion [61]. We identified one significant SNP in the intron of *ORC4* that may affect its expression or splicing, potentially altering its function in meiosis and influencing the sperm chromosomal integrity and ploidy. It may be important for proper DNA replication and meiotic division, affecting the sperm quality and causing the related disorders after cryopreservation.

Another promising candidate gene associated with sperm head injury was *GLRA3*, which encodes a subunit of the glycine receptor (GLR) involved in inhibitory neurotransmission in the central nervous system [62]. GLR on the surface of human sperm plays a role in the acrosome reaction, a critical step in fertilization [63]. This suggests that disturbances in the acrosome reaction mediated by GLRA3 may contribute to sperm head damage following cryopreservation.

We also identified significant SNPs in genes such as *TTK* (TTK protein kinase), *BCKDHB* (branched chain keto acid dehydrogenase E1 subunit beta), *PPP3CA* (protein phosphatase 3 catalytic subunit alpha), *MAP2K5* (mitogen-activated protein kinase kinase 5), *RABGAP1* (RAB GTPase activating protein 1), *EDNRA* (endothelin receptor type A), *TTC29* (tetratricopeptide repeat domain 29), *NEDD4* (NEDD4 E3 ubiquitin protein ligase), *RAD51B* (RAD51 paralog B) and *KLHL1* (kelch-like family member 1). These genes are involved in various cellular processes and pathways, such as protein phosphorylation [64], mitochondrial metabolism [65], calcium signaling [66], the acrosome reaction [67] and cytoskeletal dynamics [68]. Our findings suggest that these genes may play an important role in maintaining sperm quality and viability after cryopreservation. For example, the *TTC29* gene, encoding a tetratricopeptide repeat protein, is expressed predominantly in the testes and can modulate the assembly and functioning of flagella [69]. The *NEDD4* gene, encoding an E3 ubiquitin ligase, plays a role in mRNA metabolism and stress response [70], and its deficiency can lead to impaired spermatogenesis and male infertility. The gene for RAD51B, a member of the RAD51 family involved in DNA repair [71], may influence sperm quality by modulating meiotic recombination and DNA repair. The *KLHL1* gene, which belongs to the family of actin-organizing proteins, may be involved in cytoskeletal dynamics, actin polymerization and depolymerization [72], which could potentially affect sperm function and cryotolerance.

Our results are concordant with previous studies that have reported genetic associations with sperm head abnormalities in humans and other animals. For example, Malhotra [73] reviewed the genetic basis of sperm head defects, including globozoospermia, macrozoospermia and acephalous sperm syndrome, highlighting the role of such genes as *AURKC* (aurora kinase C), *DPY19L2* (dpy-19 like 2) and *SUN5* (Sad1 and UNC84 domain containing 5) in spermatogenesis and fertilization. These genes are involved in chromosomal segregation, sperm head formation and sperm–egg fusion, respectively, and their mutations can cause severe teratozoospermia and male infertility [73]. Yatsenko et al. [74] showed that mutations in the *ZPBP1* (zona pellucida binding protein) gene, which encodes a protein that binds to the transparent membrane of the oocyte and is involved in the recognition and fusion of sperm and egg, were found in 3.9% of infertile men with abnormal morphology of the sperm head. Therefore, *ZPBP1* may be another important candidate gene for sperm head integrity and function. The genes we identified here are also involved in various biological processes related to spermatogenesis, meiosis, cell polarity, signaling, gene expression and fertilization. Therefore, they may provide new insights into the genetic basis of sperm cryoresistance and may facilitate the development of molecular markers and strategies to improve sperm quality, eliminate sperm-related disorders and improve bovine fertility.

#### 4.1.3. Swollen Acrosomes

This study identified four genes: *LPCAT4*, *DPYD*, *SLC39A12* (solute carrier family 39 member 12) and *CACNB2* (calcium voltage-gated channel auxiliary subunit beta 2), and two miRNAs (*bta-mir-137* and *bta-mir-2420*), that were significantly associated with post-freezing acrosome swelling, a key indicator of sperm quality and fertility. LPCAT4 is a lysophosphatidylcholine acyltransferase that synthesizes phosphatidylcholine, the main component of sperm membranes [50]. Phosphatidylcholine is involved in the remodeling and stability of sperm membranes during cryopreservation [49]. We previously reported that this gene is differentially expressed in the sperm of bulls with different levels of fertility [51]. DPYD is a dihydropyrimidine dehydrogenase that catabolizes pyrimidine nucleotides, precursors for the synthesis of DNA and RNA [52]. Pyrimidine nucleotides are involved in DNA replication and repair during spermatogenesis [53]. SLC39A12 is a zinc transporter that mediates zinc uptake in cells [54]. Zinc is involved in zinc homeostasis and signaling processes in sperm [55]. This gene was previously reported to be expressed in human testes and epididymis [56]. CACNB2 is a calcium channel subunit that modulates calcium influx into cells [57]. Calcium is involved in calcium-dependent processes such as capacitation, hyperactivation [58] and the acrosome reaction of sperm [75]. The association of this gene with sperm quality traits has not been previously reported.

Thus, the above genes and miRNAs are involved in various biological processes that may influence sperm membrane integrity, DNA stability, zinc homeostasis and calcium signaling in sperm. These processes are necessary for sperm survival during cryopreservation and subsequent fertilization. Our results suggest that these genes and miRNAs may be potential candidates for improving bovine semen quality after freezing. In addition, our study is, to the best of our knowledge, the first to examine the genetic aspects associated with acrosome swelling following the freezing of animal sperm.

#### 4.1.4. Wrinkled Acrosomes

The identification of candidate genes associated with wrinkled acrosomes in Holstein bull sperm after cryopreservation is of paramount importance for understanding the underlying mechanisms affecting sperm quality and fertility. Wrinkling of the sperm acrosome, a morphological defect that impedes the acrosome reaction, can have a detrimental effect on sperm penetration into the zona pellucidum of the oocyte [76]. By investigating genomic associations with this defect, we can shed light on the genetic factors influencing acrosome biogenesis, maturation and response, which will ultimately lead to improved semen quality and increased fertility in Holstein bulls. Our study successfully identified several candidate genes that may play a critical role in the formation of wrinkled acrosomes. Among these genes, *IGFBP3* stands out as a potential regulator of sperm development and functioning. *IGFBP3* encodes a protein that modulates the activity of insulin-like growth factors (IGFs), which are known to regulate various aspects of sperm physiology, including spermatogenesis, motility, capacitation and the acrosome reaction [77,78]. By modulating the availability and activity of IGFs in the male reproductive tract, IGFBP3 may have significant effects on sperm quality and fertility.

Another candidate gene, *MPP6* (protein associated with LIN7 2, MAGUK p55 family member), belonging to the membrane-associated guanylate kinase (MAGUK) family, participates in cell adhesion [79] and signal transduction [80]. While MPP6 is involved in ensuring the stability and integrity of the sperm membrane [81], its role in acrosome wrinkling remains unclear. However, its association with 5.8S rRNA maturation [82] suggests a potential effect on the expression of acrosomal proteins, which may affect acrosome morphology. The third candidate gene, *NPY*, encodes a neuropeptide that regulates various physiological processes, including sperm motility and viability. Through its receptors on sperm, NPY can influence sperm function and fertility by modulating the activity of the sympathetic nervous system in the male reproductive tract [83]. The fourth candidate gene, *MON2*, encodes a protein involved in the organization and functioning of the Golgi complex [84], which is required for the formation and functioning of the acrosome [85]. The role of MON2 in regulating the trafficking and processing of acrosomal proteins suggests its potential influence on acrosome morphology and functionality. Finally, a fifth candidate gene, *STEAP1*, is expressed predominantly in prostate tissue and is involved in sperm maturation and capacitation [86]. By modulating metal ion homeostasis and redox status in the male reproductive tract, STEAP1 may play a role in regulating the sperm acrosome reaction.

Previous studies have also reported genomic associations with sperm acrosome defects in other animal species and humans. For example, Sironen et al. [87] identified a region on pig chromosome 15 that contained the *STK17B* (serine/threonine kinase 17b) and *HECW2* (HECT, C2 and WW domain containing E3 ubiquitin protein ligase 2) genes. It was associated with acrosome tuberculation defects, a severe form of acrosome damage that affects both acrosomal granules and chromatin. Yanagimachi [88] reviewed the molecular mechanisms of the sperm acrosome response in various animals and highlighted the role of calcium, phospholipids and SNARE proteins in the regulation of acrosome swelling and exocytosis. These factors can influence the kinetics and efficiency of the acrosome reaction and affect sperm fertility [88]. These studies suggest that there are common genetic and molecular pathways involved in acrosome biogenesis, maturation and response across species, and that the genes we identified may play conserved or differential roles in these processes.

Overall, our findings indicate the importance of these candidate genes for sperm acrosome wrinkling and their potential impact on semen quality and fertility in Holstein bulls. Further functional studies are required to elucidate the precise mechanisms by which these genes affect the morphology and functioning of the acrosome. Understanding the genetic basis of sperm acrosome wrinkling will not only increase our knowledge of sperm biology but will also enable the development of strategies to improve semen quality and fertility in Holstein bulls after cryopreservation.

### 4.2. Damaged Tails and Cell Necks (Midpieces)

The results of the current study showed that the genomic regions associated with cell neck abnormalities and damaged tails in the Holstein cattle sperm largely overlap with the genomic regions associated with head abnormalities, suggesting the presence of a common genetic basis for these defects. Among the 32 candidate genes identified for damaged cell necks and tails, 24 were also associated with head abnormalities, indicating a high degree of pleiotropy or linkage disequilibrium. These genes are involved in various biological processes such as transcriptional regulation, signal transduction, cell adhesion, cytoskeletal organization and DNA repair. Some of these genes have previously been reported to be associated with sperm morphology or fertility in cattle and other species. For example, *FOXF1* (forkhead box F1) has been associated with sperm head shape [89] in mice, *PTPRU* (protein tyrosine phosphatase receptor type U) with changes in membrane fluidity in pigs [90], and *RAD51* (RAD51 recombinase) and *ATR* (ATR serine/threonine kinase) with DNA repair and chromatin integrity in spermatogenesis [91,92].

In addition to the common candidate genes, eight genes were uniquely associated with damaged cell neck and tails and were not associated with head abnormalities. These genes include *RGS17* (regulator of G protein signaling 17), *LY86* (lymphocyte antigen 86), *NOVA1* (NOVA alternative splicing regulator 1), *SAMD5* (sterile alpha motif domain containing 5), *SASH1* (SAM and SH3 domain containing 1), *CLSPN* (claspin), *PTPRQ* (protein tyrosine phosphatase receptor type Q) and *SLC2A10* (solute carrier family 2 member 10). They may play a specific role in the development and functioning of the sperm cell necks and tails. RGS17 is a member of a family of G protein signaling regulators that modulate intracellular signaling pathways [93]. LY86 is a surface glycoprotein involved in immune responses [94]. NOVA1 is an RNA-binding protein that regulates the alternative splicing of target genes [95]. SAMD5 is a protein with a sterile alpha motif domain that may be involved in protein–protein interactions [96]. SASH1 is a scaffold protein that mediates signal transduction and cytoskeletal dynamics [97]. CLSPN is a cell cycle checkpoint protein that ensures correct chromosome segregation [98]. PTPRQ is a receptor-type protein tyrosine phosphatase that regulates cell adhesion and polarity [99]. SLC2A10 is a glucose transporter that promotes glucose uptake and metabolism [100]. The functions of these genes in abnormalities of cell necks and damaged tails of sperm require further study.

The results of the current study are consistent with previous reports of genomic associations with sperm tail and cell neck damages in cattle and other species. For example, a GWAS in Swedish Red cattle identified a frameshift mutation in the *ARMC3* (armadillo repeat containing 3) gene as a causative variant of the tail stump spermatozoa defect, characterized by a severely disorganized tail and immotile sperm [101]. *ARMC3* is one of the genes encoding axonemal proteins that form the main structure of the sperm tail. In a number of human and animal studies (as reviewed by [102]), it was also found that mutations in genes encoding components of axonemal dynein arms, such as *DNAH1*, *DNAH5*, *DNAH6*, *DNAH8* and *DNAH9* (dynein axonemal heavy chains 1, 5, 6, 8 and 9), induce multiple morphological abnormalities of flagella, a severe form of sperm neck defect characterized by short, coiled, absent or irregularly shaped flagella. According to the Lehti and Sironen [103] review of studies conducted in various mammals, it was reported that mutations in genes involved in axoneme assembly and stability, such as *SPAG6*, *SPAG16*, *SPAG17* (sperm associated antigens 6, 16 and 17) and *AKAP4* (A-kinase anchoring protein 4), cause sperm neck defects and reduced sperm motility. However, none of these genes were associated with cell neck and tail cell damage in our study, suggesting species differences in the genetic architecture of sperm neck defects. In addition, there has been reported, to the best of our knowledge, no GWAS for sperm neck disorders. Thus, the candidate genes for cell neck abnormalities and damaged tails in the Holstein cattle sperm we established here provide new insights into the genetic architecture of these disorder traits and may facilitate the development of molecular tools to improve sperm quality and enhance fertility. Further studies with larger sample sizes, more complete genome coverage, and functional validation are needed to elucidate and confirm the causal variants, molecular mechanisms and evolutionary consequences of genetic variations underlying cell neck abnormalities and damaged tails sperm in Holstein cattle and across species.

### 4.3. Total and Progressive Motility

The search for genomic associations with such traits as total and progressive motility of sperm after freezing–thawing is of great importance for understanding sperm quality and fertility in cattle. However, due to the complex nature of this trait, which is influenced by both environmental and genetic factors, identifying causative mutations and genes that determine sperm freeze–thaw tolerance has proven challenging. Sperm motility after freezing–thawing can be influenced by various factors such as age, nutrition, time of year, sperm collection method and sperm treatment. Due to the many affecting factors, identifying candidate genes is difficult. In particular, Rather et al. [104] reported the effect of the season on sperm cryotolerance and the *PEPB1* gene expression. A few other studies [1,105] showed the effect of the candidate gene *SCN8A* on cryotolerance in stallions and boars.

In our investigation, progressive sperm motility, which depends on the energy metabolism, structure, and function of sperm membranes, is a critical indicator of sperm quality and fertility. The following four genes that showed significant associations with this trait were identified: *JPH1* (junctophilin 1), *SNCAIP* (synuclein alpha interacting protein), *FSCB* and *PSMA1* (proteasome 20S subunit alpha 1). In particular, *JPH1* plays a critical role in the formation of junctional membrane complexes (JMCs), connecting the plasma membrane to the endoplasmic or sarcoplasmic reticulum in excitable cells [106]. Its involvement in calcium signaling, which is important for sperm motility and capacitation [107], is well documented [108,109,110,111]. *SNCAIP* is responsible for a protein that interacts with alpha-synuclein, which is involved in synaptic transmission and neurodegeneration [112], although its function in bovine sperm motility after cryopreservation remains unknown. *FSCB*, expressed in testes and sperm, may play a role in the late stages of fibrous membrane biogenesis [113] and sperm capacitation [114]. FSCB has been shown to inhibit the SUMOylation of ROPN1 (rhophilin associated tail protein 1) and ROPN1L (rhophilin associated tail protein 1 like), two proteins essential for sperm motility and fertility [115]. In addition, FSCB binds calcium that is essential for sperm function [116]. Studies in mice have shown that the phosphorylation of FSCB increases sperm motility [114], and the differential expression of *ROPN1* has been observed in sperm from bulls with different levels of fertility [117]. PSMA1 is involved in the proteasome synthesis and proteolytic degradation of intracellular proteins. The proteasome, along with regulatory elements, plays an important role in maintaining protein homeostasis and removing irregularly shaped or damaged proteins [118]. Interestingly, the sperm proteasome, in particular the PSMA1/α6 subunit, undergoes sperm-specific C-terminal processing, representing a unique post-translational modification [119]. The identification of these candidate genes provides new and valuable insights into the molecular mechanisms underlying progressive motility in the Holstein cattle sperm following cryopreservation.

There have been a number of studies on motility disorders. For example, in a GWAS in Italian Holstein bulls, Ramirez-Diaz et al. [34] identified 10 regions on BTA 1, 2, 4, 6, 7, 23 and 26 for total motility and eight regions on BTA 1, 2, 4, 6, 8, 16, 23 and 26 for progressive motility. Among the 150 genes for total motility and 72 genes for progressive motility identified in this study, not a single one overlapped with our candidate genes, which may indicate the breed specificity of genomic associations for these traits. It is worth noting that the genes found by Ramirez-Diaz et al. [34] were also enriched for gene ontology terms related to energy homeostasis, membrane function, sperm–egg interaction, protection against oxidative stress, olfactory receptors and the immune system. Some other studies also reported significant associations between sperm motility and genes involved in calcium signaling, proteasome function and fibrous membrane formation in different species, as we also noted. For example, in a GWAS of pig sperm traits [1], the *NME5* (NME/NM23 family member 5) gene, which regulates calcium homeostasis and sperm motility, was identified as a candidate gene for progressive motility. In another study conducted on Arctic char [120], it was found that SNPs near genes associated with proteasome activity, such as *PSMD4* (proteasome 26S subunit ubiquitin receptor, non-ATPase 4) and *PSMD14* (proteasome 26S subunit, non-ATPase 14), were associated with sperm motility after freeze–thaw. Moreover, a study of human sperm [114] showed that the fibrous membrane protein AKAP4, which interacts with FSCB, is required for sperm motility and fertility.

As a result of the present GWAS, a large number of genes were identified that affect the viability of sperm after freezing–thawing. The obtained data on associations of candidate genes with sperm disorder traits will support the development of molecular markers for assessing sperm quality and fertility. Future studies should aim to verify these associations using larger sample sizes, broader genomic coverage,, and functional validation, in order to confirm causal variants and elucidate the underlying mechanisms driving progressive motility in frozen–thawed Holstein cattle sperm.

## 5. Conclusions

Based on a comprehensive GWAS analysis approach, our findings demonstrate that cryopreservation can lead to detrimental changes in the structure and function of the acrosome, leading to its swelling, damage, loss or premature reaction. These alterations could make it more difficult for sperm to attach to, and fuse with, the egg membrane, underscoring how crucial acrosome integrity is to a healthy fertilization process. We identified significant SNPs associated with the absence of acrosomes after cryopreservation. In addition, our study identified candidate genes associated with head anomalies, damaged cell necks and tails and swollen acrosomes. These genes are also involved in various biological processes associated with sperm development and function, including transcriptional regulation, signal transduction, cell adhesion, cytoskeletal organization, DNA repair and chromatin integrity. While our research offers a crucial understanding of the genetic elements impacting sperm quality following cryopreservation, additional investigations, utilizing bigger sample sizes, more extensive genomic coverage and functional validation, are required to pinpoint causative variants and clarify the processes underlying these impacts.

Collectively, our study contributes to the understanding of the molecular mechanisms involved in sperm morphology and underlying bovine semen quality disorders after cryopreservation. The identification of genomic regions, as well as specific SNPs and candidate genes associated with post-cryopreservation acrosome integrity, head abnormalities, and damaged cell necks and tails, is shedding light on the complex genetic architecture underlying these traits. The knowledge gained here can be used to create targeted breeding strategies aimed at improving the quality of animal sperm and, as a result, increasing fertility in cattle. We thus pave the way for future research in the field of animal reproduction improvement, with further studies needed to confirm these results and explore the functional consequences of the identified genetic associations.

## Figures and Tables

**Table 1:** Localization ranges of some detected genomic associations with bull sperm disorders.

Disorders	BTA ^1^	SNP Localization, Mb	Candidate Genes
Absence of acrosomes	4	82.4–82.9	*POU6F2*, *VPS41*, *AMPH*
	11	70.9–72.5	*TCF23*, *SPDYA*, *PPP1CB*, *ABHD1*
Sperm head abnormalities	2	47.8–48.4	*ORC4*, *EPC2*, *MBD5*
Swollen acrosomes	10	28.3–28.5	*LPCAT4*, *CACNB2*
Wrinkled acrosomes	4	72.0–76.6	*IGFBP3*, *MON2*, *NPY*, *STEAP1*
Damaged sperm tails and cell necks	1	1.2–5.9	*GRIK1*, *SON*, *GART*
	2	47.7–48.4	*EPC2*, *MBD5*, *ORC4*
	5	9.6–10.3	*PTPRQ*, *ATF7IP*
	9	32.2–35.1	*MAN1A1*, *FRK*
		90.2–91.3	*RGS17*, *ESR1*
	10	14.3–14.4	*C10H15orf61*, *MAP2K5*
		80.2–80.3	*ZFYVE26*, *RAD51B*
	17	11.5–11.7	*TTC29*, *POU4F2*
	26	33.2–38.6	*ACSL5*, *RAB11FIP2*
		42.0–43.7	*SPADH2*, *GPR26*, *SPADH1*, *FGFR2*, *ATE1*

^1^ BTA, *Bos taurus* chromosome.

**Table 2:** Search for genomic associations with total and progressive motility traits after sperm cryopreservation.

SNP	BTA ^1^	SNP Position (bp)	*p*-Value	Alleles	SNP Location	Candidate Genes ^2^
Total motility						
ARS-BFGL-NGS-29995	15	38,746,059	1.40 × 10^−5^	A/G	intron variant	*PSMA1*
HAPMAP51688-BTA-115008	21	54,879,224	6.70 × 10^−6^	A/G	intergenic variant	*FSCB* (126.2 Kb), *ENSBTAG00000052854* (135.8 Kb)
Progressive motility						
BOVINEHD0400031627	4	110,457,609	9.17 × 10^−6^	T/G	intergenic variant	*ENSBTAG00000053666* (145 Kb)
BTB-00549286	7	32,855,047	8.72 × 10^−6^	G/A	intron variant	*SNCAIP*
ARS-BFGL-NGS-113271	14	39,616,301	1.22 × 10^−6^	T/C	intergenic variant	*ENSBTAG00000032944* (108.5 Kb), *JPH1* (17 Kb)

^1^ BTA, *Bos taurus* chromosome. ^2^ Distance from a significant SNP to the respective gene is given in parentheses.

## Data Availability

Data will be made accessible from corresponding authors upon reasonable request.

## Supplementary Materials

The following supporting information can be downloaded at: https://www.mdpi.com/article/10.3390/ani14020251/s1, Table S1: genomic associations with post-cryopreservation sperm head abnormalities, with regard to previously known candidate genes; Table S2: genomic associations with post-cryopreservation sperm head abnormalities, with regard to previously unknown candidate genes; Table S3: genomic associations with post-cryopreservation sperm tail and neck abnormalities, with regard to previously known candidate genes; Table S4: genomic associations with post-cryopreservation sperm tail and neck abnormalities, with regard to previously unknown candidate genes.
