# Supplementary material for: Risk of Sperm Disorders and Impaired Fertility in Frozen–Thawed Bull Semen: A Genome-Wide Association Study
Source: Animals (Basel). 2024 Jan 13;14(2):251. doi: 10.3390/ani14020251 (PMC10812825; doi:10.3390/ani14020251)
Supplement: Supplementary file 1 [file animals-14-00251-s001.zip › animals-2803040-Supplementary Table S1.pdf]

Supplementary Table S1. Genomic associations with post-cryopreservation sperm head abnormalities with regard to previously known candidate genes.

Disorders	SNP	BTA[1]	SNP position (bp)	*p*-value	Alleles	SNP location	Candidate genes[2]
Absence of acrosomes	BOVINEHD0400018765	4	68,273,671	2.17E-06	T/G	intergenic variant	*CREBP5* (70.9 Kb), *JAZF1* (171.2 Kb)
	BTB-00566744	4	82,386,574	2.89E-15	C/T	intron variant	*POU6F2*
	HAPMAP39674-BTA-65901	4	86,144,034	2.89E-15	A/C	intergenic variant	*TSPAN12* (32.9 Kb)
	HAPMAP42094-BTA-119925	4	82,923,306	2.89E-15	T/C	intergenic variant	*VPS41* (26.6 Kb), *AMPH* (111.9 Kb)
	ARS-BFGL-NGS-62112	9	103,671,451	2.89E-15	G/T	intergenic variant	*AFDN* (141.9 Kb)
	ARS-BFGL-NGS-37893	11	72,476,622	2.89E-15	A/G	upstream gene variant	*TCF23* (2.7 Kb), *ABHD1* (14.3 Kb)
	ARS-BFGL-BAC-14362	12	67,348,260	1.14E-05	T/C	intergenic variant	*GPC5* (7.5 Kb)
	BOVINEHD1100020331	11	70,961,745	2.89E-15	A/G	3′ UTR variant	*SPDYA* (9.5 Kb), *PPP1CB*
	BTA-42058-NO-RS	17	9,807,701	2.89E-15	C/T	intron variant	*NR3C2*
	HAPMAP41533-BTA-40976	17	45,029,111	2.89E-15	C/T	downstream gene variant	*ZNF26* (3.4 Kb), *ZNF84* (9.5 Kb)
	BOVINEHD2000019839	20	68,361,188	1.08E-05	G/T	intergenic variant	*U6* (55.9 Kb)
	ARS-BFGL-BAC-28344	23	4,359,518	1.09E-06	A/G	intergenic variant	*BMP5* (30 Kb)
	HAPMAP51003-BTA-58716	24	57,482,936	2.89E-15	C/T	intron variant	*ATP8B1*
	ARS-BFGL-NGS-106493	27	27,915,371	2.89E-15	C/T	intergenic variant	*NRG1* (61.4 Kb)
	ARS-BFGL-NGS-19057	29	44,196,154	1.94E-05	C/T	downstream gene variant	*CDC42EP2* (2 Kb), *DPF2* (8.2 Kb)
	ARS-BFGL-NGS-114697	2	48,356,704	5.50E-06	G/A	intron variant	*ORC4*
Sperm head disorders	HAPMAP40994-BTA-46361	19	61,630,610	5.50E-06	T/C	intergenic variant	*MAP2K6* (78.4 Kb)
	ARS-BFGL-NGS-113327	18	12,295,233	5.88E-06	C/T	intergenic variant	*FOXF1* (85.3 Kb)
	ARS-BFGL-NGS-116330	2	131,016,477	5.88E-06	A/G	intergenic variant	*ZBTB40* (76.4 Kb)
	ARS-BFGL-NGS-3988	26	41,487,372	5.88E-06	C/A	intron variant	*PTPRU*
	ARS-BFGL-NGS-57214	8	7,032,385	5.88E-06	A/G	intergenic variant	*GLRA3* (13.6 Kb)
	ARS-BFGL-NGS-83728	11	94,227,167	5.88E-06	C/T	intron variant	*RABGAP1*
	ARS-BFGL-NGS-98094	2	47,782,292	5.88E-06	C/T	intergenic variant	*EPC2* (132.3 Kb), *MBD5* (18.4 Kb)
	ARS-BFGL-NGS-98828	9	20,117,983	5.88E-06	C/T	intergenic variant	*BCKDHB* (12.3 Kb)

	BOVINEHD0600006887	6	25,017,431	5.88E-06	G/T	intron variant	*PPP3CA*
	BOVINEHD1500025946	15	65,145,149	5.88E-06	G/A	synonymous variant	*CD59*
	BTA-73210-NO-RS	5	29,544,481	5.88E-06	G/A	intron variant	*DIP2B*
	BTB-00401428	9	81,924,104	5.88E-06	A/G	intergenic variant	*AIG1* (90 Kb), *ADAT2* (16.4 Kb)
	BTB-00671456	17	11,751,720	5.88E-06	T/G	3′ UTR variant	*TTC29, POU4F2* (71 Kb)
	BTB-01560502	1	5,880,498	5.88E-06	G/A	intron variant	*GRIK1*
	HAPMAP38976-BTA-71247	10	54,582,753	5.88E-06	G/T	intron variant	*NEDD4* (*esv4011968*)
	HAPMAP41900-BTA-54636	22	46,309,192	5.88E-06	C/T	intergenic variant	*WNT5A* (195.1 Kb)
	HAPMAP45875-BTA-37931	10	80,331,159	5.88E-06	A/T	intron variant	*RAD51B*
	HAPMAP52726-RS29018162	12	44,326,840	5.88E-06	G/T	intron variant	*KLHL1*
	HAPMAP54751-RS29013947	6	19,307,772	5.88E-06	C/T	intergenic variant	*DKK2* (73.7 Kb)
	ARS-BFGL-NGS-44351	10	28,333,105	5.33E-06	T/C	intron variant	*LPCAT4*
Swollen acrosomes	ARS-BFGL-NGS-86370	3	30,756,980	6.67E-06	C/T	upstream gene variant	*PPMJ1*
	HAPMAP44656-BTA-67751	3	45,405,468	1.07E-05	C/T	intergenic variant	*DPYD* (127.5 Kb)
	ARS-BFGL-NGS-3160	10	28,548,872	1.97E-05	T/C	intergenic variant	*CACNB2* (27.3 Kb)
	HAPMAP23010-BTA-142347	4	76,657,995	1.76E-07	T/G	intergenic variant	*IGFBP3* (46.4 Kb)
Wrinkled acrosomes	BTA-97670-NO-RS	4	71,978,141	2.21E-07	A/G	intergenic variant	*NPY* (91 Kb)
	ARS-BFGL-NGS-11190	4	72,275,120	4.81E-06	T/C	intron variant	*MON2*
	HAPMAP43771-BTA-114609	4	74,823,667	9.12E-06	C/T	intergenic variant	*STEAP1* (13 Kb)

[1] BTA, *Bos taurus* chromosome. [2] Distance from a significant SNP to the respective gene is given in parentheses.
