# Supplementary material for: Risk of Sperm Disorders and Impaired Fertility in Frozen–Thawed Bull Semen: A Genome-Wide Association Study
Source: Animals (Basel). 2024 Jan 13;14(2):251. doi: 10.3390/ani14020251 (PMC10812825; doi:10.3390/ani14020251)
Supplement: Supplementary file 1 [file animals-14-00251-s001.zip › animals-2803040-Supplementary Table S2.pdf]

Supplementary Table S2. Genomic associations with post-cryopreservation sperm head abnormalities with regard to previously unknown candidate genes.

Disorders	SNP	BTA[1]	SNP position (bp)	*p*-value	Alleles	SNP location	Candidate genes[2]
Absence of acrosomes	BTA-96609-NO-RS	2	76,035,542	1.38E-05	C/T	intergenic variant	*ENSBTAT00000074814.1*
	BTB-00349550	8	57,559,835	6.08E-06	G/A	intergenic variant	*ENSBTAG00000006294* (69.6 Kb)
	ARS-BFGL-NGS-62112	9	103,671,451	2.89E-15	G/T	intergenic variant	*ENSBTAG00000052029* (87.3 Kb)
	BTB-00395187	9	59,571,491	2.89E-15	C/T	intron variant	*ENSBTAG00000049952*
	BTB-00395273	9	59,595,970	2.89E-15	G/A	intron variant	*ENSBTAG00000049952*
	ARS-BFGL-NGS-91628	12	49,371,159	5.20E-06	C/T	intergenic variant	*ENSBTAG00000054652* (15.7 Kb)
	ARS-BFGL-NGS-100023	15	1,242,901	2.89E-15	A/G	intergenic variant	*ENSBTAG00000055028* (66.8 Kb). *ENSBTAG00000046387* (60 Kb)
	BTA-21339-NO-RS	19	47,200,732	5.20E-06	C/T	intron variant	*ENSBTAG00000055001*
	BOVINEHD2000019839	20	68,361,188	1.08E-05	G/T	intergenic variant	*ENSBTAG00000045462* (48.4 Kb)
	ARS-BFGL-BAC-28344	23	4,359,518	1.09E-06	A/G	intergenic variant	*ENSBTAG00000046193* (177.4 Kb)
	HAPMAP40994-BTA-46361	19	61,630,610	5.50E-06	T/C	intergenic variant	*ENSBTAG00000049077* (34.1 Kb)
Sperm head disorders	ARS-BFGL-NGS-113327	18	12,295,233	5.88E-06	C/T	intergenic variant	*ENSBTAG00000054270* (114.6 Kb)
	ARS-BFGL-NGS-57214	8	7,032,385	5.88E-06	A/G	intergenic variant	*ENSBTAG00000053818*
	BTB-01402723	10	94,482,815	5.88E-06	A/T	intron variant	*ENSBTAG00000050021*
	HAPMAP39869-BTA-78346	10	14,327,969	5.88E-06	T/C	upstream gene variant	*C10H15orf61*
	HAPMAP41900-BTA-54636	22	46,309,192	5.88E-06	C/T	intergenic variant	*ENSBTAG00000049764* (47.7 Kb)
	HAPMAP44656-BTA-67751	3	45,405,468	1.07E-05	C/T	intergenic variant	*bta-mir-137* (38.2 Kb)
Swollen acrosomes	HAPMAP23010-BTA-142347	4	76,657,995	1.76E-07	T/G	intergenic variant	*bta-mir-2420* (51.8 Kb)
Wrinkled acrosomes							

[1] BTA, *Bos taurus* chromosome. [2] Distance from a significant SNP to the respective gene is given in parentheses.
