# Supplementary material for: Risk of Sperm Disorders and Impaired Fertility in Frozen–Thawed Bull Semen: A Genome-Wide Association Study
Source: Animals (Basel). 2024 Jan 13;14(2):251. doi: 10.3390/ani14020251 (PMC10812825; doi:10.3390/ani14020251)
Supplement: Supplementary file 1 [file animals-14-00251-s001.zip › animals-2803040-Supplementary Table S3.pdf]

Supplementary Table S3. Genomic associations with post-cryopreservation sperm tail and neck abnormalities with regard to previously known candidate genes.

SNP	BTA[1]	SNP position (bp)	*p*-value	Alleles	SNP location	Candidate genes[2]
ARS-BFGL-NGS-113327	18	12,295,233	3.50E-20	C/T	intergenic variant	*FOXF1* (84.6 Kb)
ARS-BFGL-NGS-116330	2	131,016,477	3.50E-20	A/G	intergenic variant	*ZBTB40* (76.4 Kb), *WNT4* (28.6 Kb)
ARS-BFGL-NGS-83728	11	94,227,167	3.50E-20	C/T	intron variant	*RABGAP1*
ARS-BFGL-NGS-98094	2	47,782,292	3.50E-20	C/T	intergenic variant	*EPC2* (132.3 Kb), *MBD5* (18.4 Kb)
ARS-BFGL-NGS-98828	9	20,117,983	3.50E-20	C/T	intergenic variant	*TTK* (42.3 Kb), *BCKDHB* (12.3 Kb)
BOVINEHD0600006887	6	25,017,431	3.50E-20	G/T	intron variant	*PPP3CA*
BOVINEHD1000004800	10	14,392,278	3.50E-20	A/G	intron variant	*MAP2K5*
BOVINEHD1500025946	15	65,145,149	3.50E-20	G/A	synonymous variant	*CD59*
BTA-73210-NO-RS	5	29,544,481	3.50E-20	G/A	intron variant	*DIP2B*
BTB-00401428	9	81,924,104	3.50E-20	A/G	intergenic variant	*AIG1* (90 Kb), *ADAT2* (16.4 Kb)
BTB-00671456	17	11,751,720	3.50E-20	T/G	3′ UTR variant	*TTC29*, *POU4F2* (71 Kb)
BTB-01435781	17	11,466,582	3.50E-20	C/T	intergenic variant	*TTC29* (13.6 Kb)
BTB-01560502	1	5,880,498	3.50E-20	G/A	intron variant	*GRIK1*
HAPMAP38976-BTA-71247	10	54,582,753	3.50E-20	G/T	intron variant	*NEDD4* (*esv4011968*)
HAPMAP39869-BTA-78346	10	14,327,969	3.50E-20	T/C	upstream gene variant	*C10H15orf61*
HAPMAP41900-BTA-54636	22	46,309,192	3.50E-20	C/T	intergenic variant	*WNT5A* (195.1 Kb)
HAPMAP45875-BTA-37931	10	80,331,159	3.50E-20	A/T	intron variant	*RAD51B*
HAPMAP52726-RS29018162	12	44,326,840	3.50E-20	G/T	intron variant	*KLHL1*
HAPMAP54751-RS29013947	6	19,307,772	3.50E-20	C/T	intergenic variant	*DKK2* (73.7 Kb)
BTB-00403297	9	91,346,194	1.67E-07	A/C	intron variant	*RGS17*
HAPMAP44674-BTA-84638	9	91,158,204	1.67E-07	G/T	intron variant	*RGS17*
BTA-122302-NO-RS	23	48,444,673	3.34E-07	T/G	intergenic variant	*LY86* (11.6 Kb), *F13A1* (186.9 Kb)
ARS-BFGL-NGS-107194	3	110,642,401	6.88E-07	T/C	intergenic variant	*CLSPN* (10.9 Kb), *C3H1orf216* (8.2 Kb)
BTB-00222372	5	10,278,129	7.95E-07	C/T	intron variant	*PTPRQ*
BTB-00894494	13	76,201,517	7.95E-07	A/C	intergenic variant	*SLC2A10* (96.2 Kb), *U6* (16.4 Kb)
ARS-BFGL-NGS-114697	2	48,356,704	9.56E-07	G/A	intron variant	*ORC4*
HAPMAP40994-BTA-46361	19	61,630,610	9.56E-07	T/C	intergenic variant	*MAP2K6* (78.4 Kb)
BTA-74596-NO-RS	5	96,166,308	1.11E-06	G/A	intergenic variant	*ATF7IP* (25.1 Kb)
BTB-00389188	9	35,109,955	1.11E-06	C/T	intron variant	*FRK*
BTB-00880226	4	89,299,743	1.11E-06	G/A	intergenic variant	*TMEM229A* (62.8 Kb)

ARS-BFGL-NGS-104332	7	26,829,547	1.33E-06	A/G	intron variant	*FBN2*
ARS-BFGL-NGS-46105	9	90,247,980	1.39E-06	A/T	intron variant	*ESR1*
BTA-107369-NO-RS	8	101,683,774	1.39E-06	A/G	intron variant	*SVEP1*
ARS-BFGL-BAC-6557	1	1,234,172	1.44E-06	C/A	3′ UTR variant	*SON*
ARS-BFGL-NGS-108850	26	42,896,381	1.44E-06	CTG/-	intergenic variant	*SPADH2* (9.5 Kb), *SPADH1* (17 Kb)
ARS-BFGL-BAC-12931	1	1,288,510	1.51E-06	A/G	intron variant	GART
ARS-BFGL-NGS-19833	4	113,416,523	2.37E-06	T/C	intergenic variant	*KRBA1* (20.4 Kb), *SSPO* (8.8 Kb)
HAPMAP24713-BTA-61641	26	42,033,314	3.33E-06	A/G	intergenic variant	*FGFR2* (102.3 Kb), *ATE1* (7.5 Kb)
ARS-BFGL-NGS-20623	26	43,687,318	4.52E-06	T/C	intergenic variant	*GPR26* (63.4 Kb)
ARS-BFGL-NGS-48262	26	33,191,302	4.52E-06	C/G	intron variant	*ACSL5*
BTA-61502-NO-RS	26	38,585,670	4.52E-06	G/A	intergenic variant	*RAB11FIP2* (27.3 Kb)
ARS-BFGL-NGS-96337	10	80,223,217	5.15E-06	C/A	intron variant	*ZFYVE26*
HAPMAP42436-BTA-114985	24	25,895,037	6.27E-06	T/C	intergenic variant	*TTR* (30.7 Kb), *DSG2* (19.1 Kb)
BTA-35174-NO-RS	14	63917588	8.14E-06	A/G	intergenic variant	*KLF10* (34.8 Kb)
BTA-50305-NO-RS	1	127568464	8.15E-06	G/A	intron variant	*ATR*
HAPMAP52436-RS29009653	6	99732094	8.15E-06	G/T	intron variant	*PLAC8A-202*
BOVINEHD0200036990	2	127435919	8.19E-06	T/C	intron variant	*CEP85*
BTB-01632102	9	32171013	9.78E-06	T/C	intron variant	*MAN1A1*

[1] BTA, *Bos taurus* chromosome. [2] Distance from a significant SNP to the respective gene is given in parentheses.
