# Supplementary material for: Risk of Sperm Disorders and Impaired Fertility in Frozen–Thawed Bull Semen: A Genome-Wide Association Study
Source: Animals (Basel). 2024 Jan 13;14(2):251. doi: 10.3390/ani14020251 (PMC10812825; doi:10.3390/ani14020251)
Supplement: Supplementary file 1 [file animals-14-00251-s001.zip › animals-2803040-Supplementary Table S4.pdf]

Supplementary Table S4. Genomic associations with post-cryopreservation sperm tail and neck abnormalities with regard to previously unknown candidate genes.

SNP	BTA[1]	SNP position (bp)	*p*-value	Alleles	SNP location	Candidate genes[2]
ARS-BFGL-NGS-113327	18	12,295,233	3.50E-20	C/T	intergenic variant	*ENSBTAG00000054270* (113.9 Kb)
BOVINEHD2400014613	24	51,876,107	3.50E-20	C/T	intergenic variant	*ENSBTAG00000051721* (105 Kb), *ENSBTAG00000049503* (54.6 Kb)
BTB-01402723	10	94,482,815	3.50E-20	A/G	intron variant	*ENSBTAG00000050021*
HAPMAP39869-BTA-78346	10	14,327,969	3.50E-20	T/C	upstream gene variant	*C10H15orf61*
HAPMAP41900-BTA-54636	22	46,309,192	3.50E-20	C/T	intergenic variant	*ENSBTAG00000049764* (47.7 Kb)
HAPMAP39377-BTA-113470	21	36,623,599	4.84E-07	A/C	intergenic variant	*ENSBTAG00000054263* (66.2 Kb)
ARS-BFGL-NGS-107194	3	110,642,401	6.88E-07	T/C	intergenic variant	*C3H1orf216* (8.2 Kb)
HAPMAP40994-BTA-46361	19	61,630,610	9.56E-07	T/C	intergenic variant	*ENSBTAG00000049077* (34.1 Kb)
BTA-74596-NO-RS	5	96,166,308	1.11E-06	G/A	intergenic variant	*ENSBTAG00000051346* (93.4 Kb)
HAPMAP48384-BTA-54234	22	35,354,500	1.44E-06	G/A	intergenic variant	*ENSBTAG00000051801* (17.7 Kb)
BTA-61502-NO-RS	26	38,585,670	4.52E-06	G/A	intergenic variant	*ENSBTAG00000049634* (199.2 Kb)
BTA-35174-NO-RS	14	63,917,588	8.14E-06	A/G	intergenic variant	*ENSBTAG00000052148* (58.7 Kb)
ARS-BFGL-NGS-18962	1	130,517,998	8.15E-06	T/C	synonymous variant	*ENSBTAG00000051936*
ARS-BFGL-NGS-73903	23	49,725,139	8.19E-06	A/G	intergenic variant	*ENSBTAG000000513166* (115.3 Kb), *ENSBTAG00000052017*

[1] BTA, *Bos taurus* chromosome. [2] Distance from a significant SNP to the respective gene is given in parentheses.
